# Identification of key genes and pathways involved in response to pain in goat and sheep by transcriptome sequencing

**DOI:** 10.1186/s40659-018-0174-7

**Published:** 2018-08-17

**Authors:** Xiuling Deng, Dong Wang, Shenyuan Wang, Haisheng Wang, Huanmin Zhou

**Affiliations:** 1 College of life Science, Inner Mongolia Agricultural University, No. 306 Zhaowuda Road, Saihan District, Hohhot, 010018 People’s Republic of China; 2 College of Basic Medicine, Inner Mongolia Medical University, Hohhot, 010110 People’s Republic of China; 3 Neurology Department, Inner Mongolia People’s Hospital, Hohhot, 010017 People’s Republic of China

**Keywords:** Chronic pain, Transcriptome sequencing, Differentially expressed genes, Gene ontology, Goat, Sheep

## Abstract

**Purpose:**

This aim of this study was to investigate the key genes and pathways involved in the response to pain in goat and sheep by transcriptome sequencing.

**Methods:**

Chronic pain was induced with the injection of the complete Freund’s adjuvant (CFA) in sheep and goats. The animals were divided into four groups: CFA-treated sheep, control sheep, CFA-treated goat, and control goat groups (n = 3 in each group). The dorsal root ganglions of these animals were isolated and used for the construction of a cDNA library and transcriptome sequencing. Differentially expressed genes (DEGs) were identified in CFA-induced sheep and goats and gene ontology (GO) enrichment analysis was performed.

**Results:**

In total, 1748 and 2441 DEGs were identified in CFA-treated goat and sheep, respectively. The DEGs identified in CFA-treated goats, such as C-C motif chemokine ligand 27 (CCL27), glutamate receptor 2 (GRIA2), and sodium voltage-gated channel alpha subunit 3 (SCN3A), were mainly enriched in GO functions associated with *N*-methyl-d-aspartate (NMDA) receptor, inflammatory response, and immune response. The DEGs identified in CFA-treated sheep, such as gamma-aminobutyric acid (GABA)-related DEGs (gamma-aminobutyric acid type A receptor gamma 3 subunit [GABRG3], GABRB2, and GABRB1), SCN9A, and transient receptor potential cation channel subfamily V member 1 (TRPV1), were mainly enriched in GO functions related to neuroactive ligand-receptor interaction, NMDA receptor, and defense response.

**Conclusions:**

Our data indicate that NMDA receptor, inflammatory response, and immune response as well as key DEGs such as CCL27, GRIA2, and SCN3A may regulate the process of pain response during chronic pain in goats. Neuroactive ligand-receptor interaction and NMDA receptor as well as GABA-related DEGs, SCN9A, and TRPV1 may modulate the process of response to pain in sheep. These DEGs may serve as drug targets for preventing chronic pain.

**Electronic supplementary material:**

The online version of this article (10.1186/s40659-018-0174-7) contains supplementary material, which is available to authorized users.

## Background

Chronic pain is considered as a major physical and mental health problem. Millions of people are affected by uncomfortable conditions such as back pain, headache, and arthritis [1, 2]. Chronic pain is often difficult to treat [3, 4], and 60% of patients with chronic pain experience pain after 1 year of treatment [5]. Although extensive research has been conducted on the genetics of chronic pain, the mechanism underlying chronic pain is largely unknown owing to the involvement of several genes [6]. For a better understanding of the etiology and treatment of chronic pain, it is imperative to further elucidate the key mechanism.

Dorsal root ganglion (DRG) contains many primary sensory neurons that are responsible for the transduction and modulation of sensory information and its transmission to the spinal cord, an active participant in the development of chronic neuropathic pain [7]. Accumulating evidence has confirmed that DRG plays a critical role in the induction and maintenance of chronic pain [8]. DRG is involved in the transduction of pain to the central nervous system and exhibits various pathophysiologic changes during chronic pain [9]. Moreover, DRG stimulation in response to peripheral afferent fiber injury may induce multiple abnormal changes that occur within the DRG and stabilize or reduce the hyperexcitability of DRG neurons and consequently decrease chronic neuropathic pain [10]. Furthermore, DRG stimulation is considered as a promising treatment strategy to offer relief from chronic pain, and DRG therapeutics have been implicated for the treatment of chronic pain [11, 12]. KCNQ channels are found expressed in nociceptive DRG neurons and their activation is effective in reducing chronic pain [13]. The differential expression of ATP-gated P2X receptors in DRG is shown to play important roles in the regulation of nociceptive mechanisms in chronic neuropathic pain and visceralgia rat models [14]. The transient receptor potential melastatin 8 in DRG plays a key role in chronic neuropathic pain [15]. Microarray analysis of DRG gene expression has been used for the identification of key cytokines involved in the regulation of chronic pain [16]. Despite these advances, the regulatory mechanisms mediating the development of chronic pain are largely unknown. Given that the spinal DRG is the primary center for the conduction and maintenance of pain, studies on DRG transcriptome may be helpful for the comprehensive and systematic elucidation of the key regulatory mechanisms underlying chronic pain.

In the present study, chronic pain was induced by inoculation of the complete Freund’s adjuvant (CFA) in sheep and goats, as animals injected with CFA are known to quickly develop hyperalgesia and have low latency times on their inflamed paws [17]. A clear advantage of using a large animal model such as sheep is the similarity in the body size, spine dimensions, and cardiac and pulmonary functional parameters to humans [18, 19]. Another benefit of using sheep as a chronic pain model is the longer lifespan of sheep (approximately 20 years) than rats (about 2–4 years) [20]. Previous studies of sheep foot rot have revealed a chronic inflammatory pain condition [21, 22]. Our previous observation on the differences in responses to pain between sheep and goats encouraged us to select sheep and goats as the animal models. After CFA inoculation, we analyzed the pain response mechanism in goats and sheep through transcriptome sequencing using the DRG tissues and performed bioinformatic analysis. Our findings may improve the understanding of the molecular mechanisms underlying chronic pain and provide valuable information for the development of treatment strategy.

## Methods

### Animal preparation, treatment, and grouping

All experiments were approved by the local animal care and use committee. Six sheep and six goats weighing 30–40 kg and 2- to 3-year old were obtained from the key laboratory of Inner Mongolia Autonomous Region (Hohhot, Inner Mongolia, China) and housed in the central housing facility under a standard condition. Food and water were available ad libitum. The CFA-induced arthritic rat model has been used to study chronic pain [23]; hence, chronic pain was induced by CFA inoculation in the sheep and goats. CFA (Sigma, USA) is a mixture of heat-killed *Mycobacterium tuberculosis* and paraffin oil at a concentration of 1 mg/mL. Prior to experiments, CFA was diluted 1:1 in 0.9% sterile saline. Each sheep or goat was injected in the left footpad (hind paw) with 1 mL of CFA under isoflurane anesthesia. This dose was chosen for the following reasons: After the injection of 1 mL of CFA, pain responses such as redness, swelling, and hyperalgesia were markedly observed at the injection site in both sheep and goats, indicating that the inflammatory pain was successfully induced by CFA; however, the induction effect was not satisfactory under or above this dose of CFA. Sheep or goats injected with 1 mL of saline served as controls. The animals were divided into four groups, namely, CFA-treated sheep (st1-3), control sheep (sc1-3), CFA-treated goat (gt1-3), and control goat (gc1-3) groups (n = 3 in each group).

### Isolation of DRG

After 72 h from CFA inoculation, the animals were sequentially slaughtered under the anesthesia of Deer Sleeping Ling. This time point was selected for the following reasons: (1) From 6 to 72 h after the injection of CFA, the redness and swelling at the injection sites in the left footpads of goats and sheep gradually aggravated. The animals showed signs of severe pain with different degrees of functional abnormalities such as tiptoe, lameness, and inability to stand at 72 h of injection, and these symptoms began to ease after 96 h of injection. (2) The time point of 72 h is long enough for the development of changes in the transcription/translation of proteins that are involved in the sensory neuron response to inflammation [24]. The lumbar spinal bone was placed on the ice and both sides of the muscles were removed under sterile conditions. The spinous process and transverse process of spines were fully exposed and cut-off for the isolation of the spinal cord. At the spinal canal of the posterior root of the spinal nerve, DRG was stripped-off and the L3-L5 DRG was collected. After rinsing in the RNA protection solution, the L3-L5 DRG was quickly stored in liquid nitrogen.

### Construction of the cDNA library of DRGs and high-throughput sequencing

Total RNA was extracted from DRGs using TRIzol regents and treated with RQ1 DNase (Promega) to remove DNA. The concentration and purity of the extracted RNA was determined by measuring the ratio of the absorbance at 260 and 280 nm (A260/A280) using SmartSpec plus (Bio-Rad Laboratories, Hercules, Calif). The integrity of the extracted RNA was confirmed with 1.5% agarose gel electrophoresis. A total of 10 μg of total RNA was used for RNA-seq library preparation. For directional RNA-seq library preparation, polyadenylated mRNAs were purified with oligo(dT)-conjugated magnetic beads (Invitrogen) and iron fragmented at 95 °C. After end repair and 5′ adaptor ligation, the cDNAs were reverse transcribed, purified, and amplified. The PCR products (200–500 bp) were finally purified and stored at − 80°C for subsequent high-throughput sequencing. The cDNA libraries were prepared and applied onto Illumina Hiseq 2000 system for 100 nucleotide pair-end sequencing by Majorbio. Inc (Shanghai, China).

### Preprocessing and quality control of sequencing data

Pre-processing for the retrieval of the clean reads was performed as follows: The reads with two N were excluded; the adapter sequences were removed according to the joint information; reads with low Q < 16 were removed. The quality assessment of the raw reads was conducted using FASTQC (http://www.bioinformatics.babraham.ac.uk/projects/fastqc/).

### Read mapping and transcription annotation

The clean reads were mapped to the reference genome of sheep (ftp://ftp.ncbi.nlm.nih.gov/genomes/Ovis_aries/) and goat (http://goat.kiz.ac.cn/GGD) using TopHat2.0.1 [25] with default parameters. Bowtie was used during mapping, and no more than one mismatched read was allowed. For calculating the gene expression, the mRNA length and sequencing depth were homogenized using reads per kilo base of a gene per million reads (RPKM) algorithm.

### Correlation analysis for the gene expression levels in different samples

Correlation analysis for gene expression levels between any two samples was performed for the three duplicate samples of sheep/goat under the same treatment condition to avoid any individual differences. If the correlation coefficient (R) was close to 1, the expression patterns between different samples were considered to have higher similarity, showing that the data had high degree of homogenization and were reliable for the subsequent analysis. In our study, the sample was deleted if R^2^ > 0.8. To further avoid any individual difference, clustering heatmap analysis for the RPKM data was conducted using the pheatmap version 1.0.8 (http://cran.r-project.org/web/packages/pheatmap/index.html) in R3.4.1.

### Identification of differentially expressed genes (DEGs)

We identified DEGs in the CFA-treated sheep group and the control sheep group using the likelihood ratio tests in edgeR package [26] in R language. The cut-off thresholds were fold change (FC) ≥ 2 or < 0.5 and P ≤ 0.01. Moreover, all gene sequences of goats were annotated by blasting to the sheep genome sequences. The DEGs in the CFA-treated goat group were compared with those in the control goat group using the edgeR package with the same thresholds. Moreover, the common DEGs (co-DEGs) between the CFA-treated goat and sheep groups were identified and visualized by Venn diagram.

### Gene ontology (GO) enrichment analysis

GO (http://www.geneontology.org) [27] is used for exploring the functions of large-scale genomic or transcriptomic data, and mainly includes biological process (BP), molecular function (MF), and cellular component (CC). GO enrichment analyses for DEGs in different groups were performed using an online tool database for Annotation, Visualization, and Integrated Discovery (DAVID, http://david.abcc.ncifcrf.gov/) [28]. To further obtain the significant GO functions, the enriched GO terms were clustered using the functional annotation clustering tool in DAVID, wherein the enrichment score for each cluster was calculated as the negative log of the geometric mean of P values in the cluster. Each functional cluster contained GO functions with similar biological meaning, owing to the presence of similar gene members. The more the enrichment score, the more significant was the cluster. Enrichment score > 0.5 was set as the cut-off value.

## Results

### High-throughput sequencing data

The results showed that the A260/A280 of extracted total RNA from all DRGs samples was 1.8–2.1, indicating that total RNA from all DRGs samples could be used for construction of the cDNA library of DRGs and subsequent high-throughput sequencing. Using the Illumina sequencing technology, more than 41,000,000 clean reads were obtained from each sample. The GC content of these 12 samples was all less than 50 and Q30(%) of these samples was about 90%, indicating that the obtained data were good and reliable for the subsequent analysis.

According to the sequencing data, a total of 20,951 genes were identified from six goat samples, accounting for 97.95% (20,951/21,389) of all goat genes. In addition, 22,066 genes were identified from six sheep samples, accounting for 88.15% (22,066/25,033) of all sheep genes. These data indicate that most of genes were expressed in DRG tissues.

### Correlation analysis for the gene expression levels in different samples

**Fig. 1:**
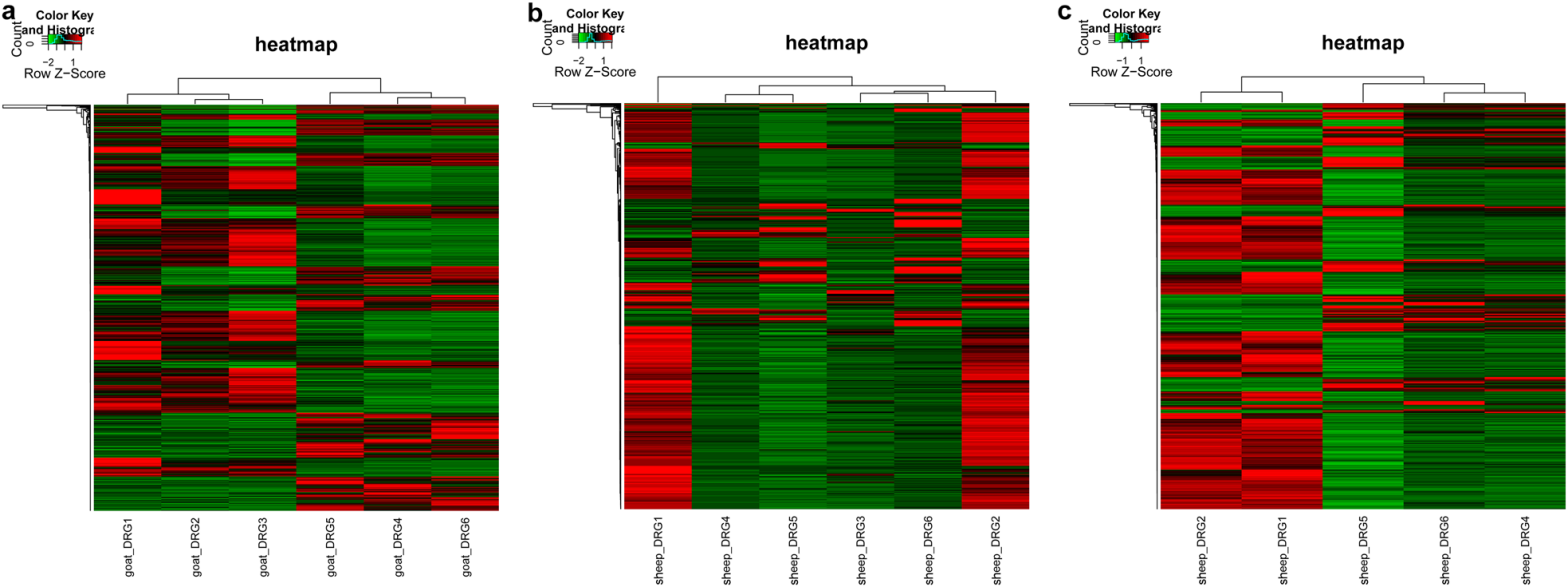
Heatmap analysis of goat (**a**) and sheep (**b**, **c**) samples. The below abscissa axis represents samples. Red shows upregulated genes, while green shows downregulated genes. In (**c**) sc3 sample was detected

To avoid the individual difference, correlation analysis for gene expression levels between any two samples was performed (data not shown). The results revealed the high correlation between any two samples in the control goat or/and CFA-treated goat groups (all R^2^ > 0.92), indicating that the individual difference was small. Furthermore, high correlation was observed between any two samples in the control sheep or/and CFA-treated sheep groups (all R^2^ > 0.8). High correlations were also observed between control goat and sheep samples as well as between CFA-treated goat and sheep samples (all R^2^ > 0.89), indicating that the expression patters of common genes in goat and sheep were similar. Heatmap analysis results showed that the control and CFA-treated goat samples were clustered (Fig. 1a), indicating that CFA treatment induced changes in the expression of goat genes. In addition, sc3 were clustered together with three CFA-treated sheep samples (Fig. 1b), suggesting that sc3 samples had larger individual differences with other two control sheep samples. The sc3 sample was detected and was not used for subsequent analyses. After the detection of the sc3 sample, the results of the hierarchical clustering analysis showed that the control and CFA-treated sheep samples were clustered (Fig. 1c).

### Identification of DEGs

**Fig. 2:**
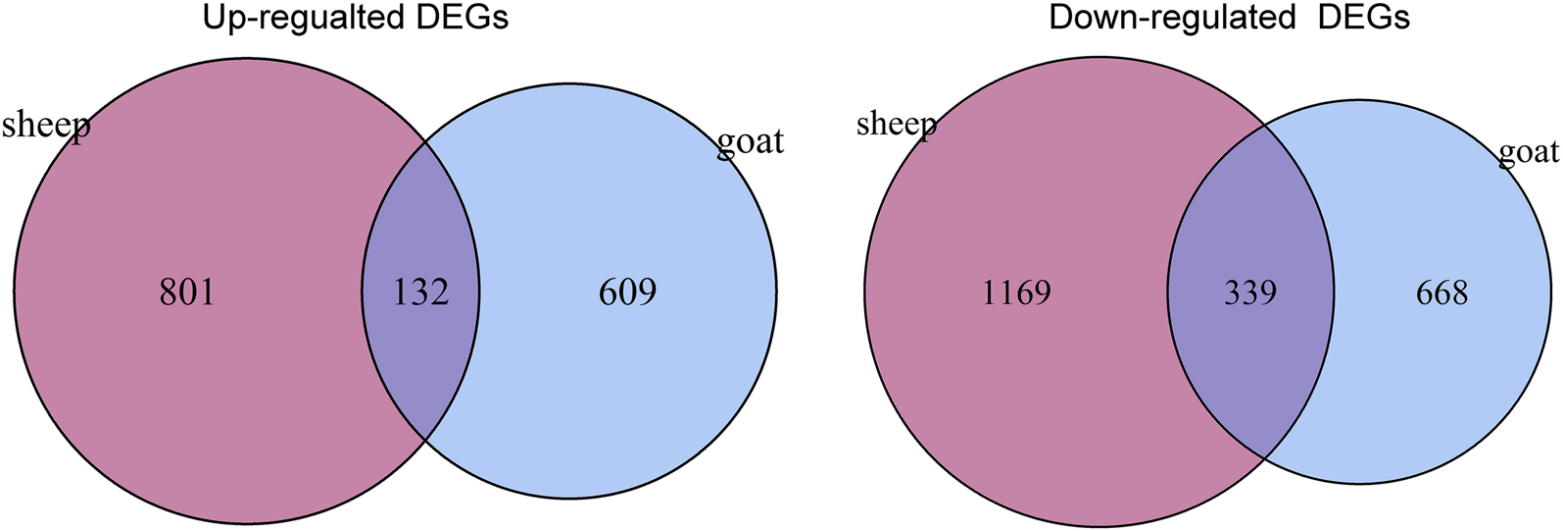
Venn diagram showed the common differentially expressed genes (co-DEGs) between the CFA-treated goat and sheep groups as compared with the control sheep and goat groups

Using the edgeR package, a total of 1748 DEGs (741 upregulated and 1007 downregulated) were identified in the CFA-treated goat group as compared with the control goat group. In addition, 2441 DEGs (993 upregulated and 1508 downregulated) were identified in the CFA-treated sheep group as compared with the control sheep group. A total of 471 common DEGs (132 upregulated and 339 downregulated) were screened out from the CFA-treated goat and sheep groups as compared with the control sheep and goat groups (Fig. 2). Furthermore, 2953 DEGs (1762 upregulated and 1173 downregulated) were identified between the control sheep and goat groups.

### Functional analysis for DEGs

**Fig. 3:**
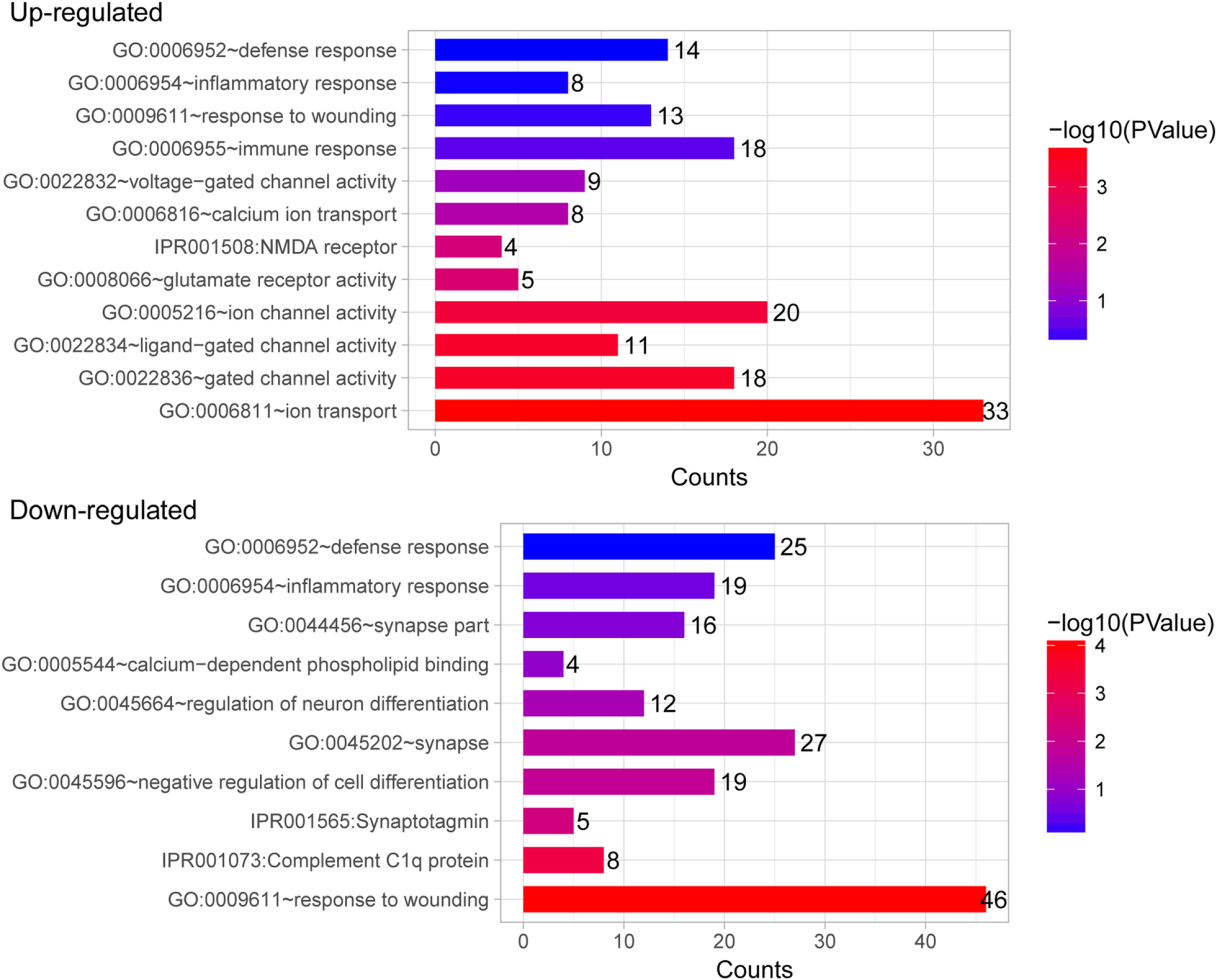
The significantly enriched gene ontology terms associated with pain in goats

**Fig. 4:**
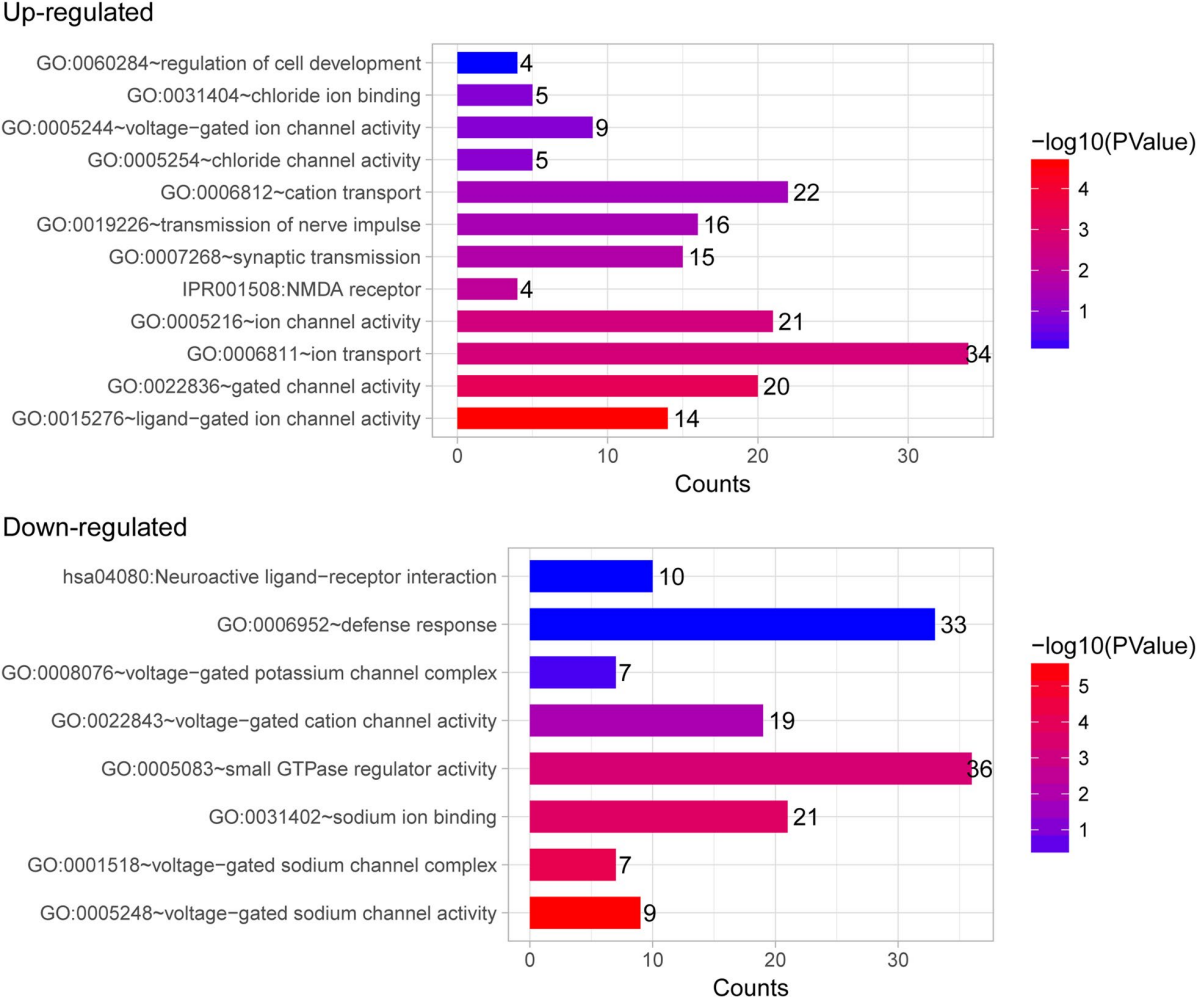
The significantly enriched gene ontology terms associated with pain in sheep

To better understand the function of DEGs, GO functional enrichment analysis was performed. DEGs identified in the CFA-treated goats were mainly enriched in GO functions associated with gated channel activity, N-methyl-d-aspartate (NMDA) receptor, inflammatory response, immune response, and defense response (Table 1 and Fig. 3). Key DEGs such as C-C motif chemokine ligand 27 (CCL27), glutamate receptor 2 (GRIA2), glutamate ionotropic receptor NMDA type subunit 2A (GRIN2A), calcium voltage-gated channel subunit alpha 1E (CACNA1E), sodium voltage-gated channel alpha subunit 3 (SCN3A), and potassium two pore domain channel subfamily K member 1 (KCNK1) were enriched in these GO functions and may play key roles in the process of response to pain in goats (Table 1). Moreover, the DEGs identified in the CFA-treated sheep were mainly enriched in GO functions related to gated channel activity, neuroactive ligand-receptor interaction, NMDA receptor, voltage-gated potassium channel complex, and defense response (Table 2 and Fig. 4). Many gamma-aminobutyric acid (GABA)-related DEGs such as gamma-aminobutyric acid type A receptor gamma 3 subunit (GABRG3), gamma-aminobutyric acid type A receptor beta 2 subunit (GABRB2), and gamma-aminobutyric acid type A receptor beta 1 subunit (GABRB1) were markedly upregulated. In addition, SCN9A and transient receptor potential cation channel subfamily V member 1 (TRPV1) were found markedly downregulated. These DEGs may be involved in the process of response to pain in sheep. GO clusters enriched by DEGs identified between CFA-treated animals and control animals are shown in Additional file 1: Table S1.

**Table 1:** The significantly enriched GO terms associated with pain in goats

Go term	Count	P value	Gene
Up-regulated			
GO:0006811 ~ ion transport	33	1.69E−04	SCN3A, SLC38A8, GABRB1, GRIK3, CNGB1, SLCO1A2, GRID2, SLC4A4, KCNG3, CHRNA1, CLCA1, GABRA4, TRPC5, SLC12A3, GRIN2A, SLC22A20, KCNK1, CACNA2D4, ITPR2, SLCO1B3, GRIA2, SLC17A4, SLC26A7, CACNA1E, KCNH8, ATP7B, KCNH5
GO:0022836 ~ gated channel activity	18	3.76E−04	GABRA4, SCN3A, TRPC5, GABRB1, GRIK3, GRIN2A, CNGB1, KCNK1, ITPR2, CACNA2D4, GRIA2, GRID2, KCNH8, CACNA1E, KCNG3, CHRNA1, FGF2, KCNH5
GO:0022834 ~ ligand-gated channel activity	11	3.98E−04	GRIA2, GABRA4, TRPC5, GABRB1, GRIK3, GRID2, GRIN2A, CNGB1, CHRNA1, KCNK1, ITPR2
GO:0005216 ~ ion channel activity	20	6.64E−04	CLCA1, GABRA4, SCN3A, TRPC5, GRIK3, GABRB1, GRIN2A, CNGB1, KCNK1, ITPR2, CACNA2D4, GRIA2, SLC26A7, GRID2, KCNH8, CACNA1E, KCNG3, CHRNA1, FGF2, KCNH5
GO:0022832 ~ voltage-gated channel activity	9	0.058679	SCN3A, GRIN2A, CACNA1E, KCNH8, KCNG3, KCNK1, FGF2, KCNH5, CACNA2D4
GO:0008066 ~ glutamate receptor activity	5	0.004105	GRM3, GRIA2, GRIK3, GRID2, GRIN2A
IPR001508:NMDA receptor	4	0.005749	GRIA2, GRIK3, GRID2, GRIN2A
GO:0006816 ~ calcium ion transport	8	0.03123	CLCA1, NPY, TRPC5, GRIN2A, CACNA1E, JPH1, ITPR2, CACNA2D4
GO:0006955 ~ immune response	18	0.291463	CXCL1, PSMB10, AQP9, S100A7, C6, CXCL9, CD180, PDCD1LG2, CCL27, TNFSF8, APOA4, C8A, CD96, OASL, CFHR5, VSIG4, IL1RAPL1, IFI6
GO:0009611 ~ response to wounding	13	0.432142	CXCL1, SELP, C6, MAP1B, CXCL9, GRIN2A, CD180, C8A, FGA, SERPINB2, VSIG4, CFHR5, FGF2
GO:0006954 ~ inflammatory response	8	0.526807	C8A, CXCL1, SELP, C6, CXCL9, CFHR5, VSIG4, CD180
GO:0006952 ~ defense response	14	0.540829	CXCL1, SELP, S100A7, C6, CXCL9, FOXN1, VARS, CD180, CLEC1A, APOA4, C8A, VSIG4, CFHR5, IL1RAPL1
Down-regulated			
IPR001073:Complement C1q protein	8	5.07E−04	CBLN4, C1QTNF3, C1QTNF1, C1QTNF2, EMILIN2, MMRN1, ADIPOQ, COL8A2
GO:0009611 ~ response to wounding	46	8.77E−05	GGCX, ERBB3, F13A1, TGFB3, AFAP1L2, MMRN1, SRF, TIMP3, SHH, AZU1, CCL24, IGSF10, S1PR3, HRH1, CASP3, CCL21, LTB4R, LOX, THBS1, FN1, RAB27A, INA, KLF6, CR2, LIPA, GATM, CYP1A1, PDPN, SAAL1, ANXA1, CHST3, COL5A1, NOTCH3, TNFAIP6, SDC1, LYVE1, THBD, TFRC, ADM, SERPINF2, ITGA5, PDGFRA, HBEGF, VCAN, IGFBP4, AOC3
GO:0006954 ~ inflammatory response	19	0.270975	LIPA, CR2, PDPN, SAAL1, ANXA1, AFAP1L2, CCL24, AZU1, S1PR3, TNFAIP6, HRH1, TFRC, LTB4R, CCL21, SERPINF2, THBS1, IGFBP4, AOC3, FN1
GO:0006952 ~ defense response	25	0.855432	AFAP1L2, CCL24, AZU1, S1PR3, HRH1, LTB4R, CCL21, THBS1, FN1, RAB27A, CR2, LIPA, PDPN, SAAL1, ANXA1, COLEC12, INHBB, TNFAIP6, INHBA, BPI, TFRC, SERPINF2, IGFBP4, ICOSLG, AOC3
GO:0045202 ~ synapse	27	0.015927	CDK5R1, SNCAIP, SYT4, ERBB3, SYT6, SV2B, PRKACA, SV2A, APBA1, GABRG2, EFNB1, SYT11, RIMBP2, SYT12, NLGN1, LIN7C, PPP1CC, RPH3A, SSPN, CBLN4, EGFLAM, COLQ, MAP1S, GRM8, CHRNB3, VAMP3, DOC2B
GO:0044456 ~ synapse part	16	0.180734	GABRG2, SNCAIP, SYT4, ERBB3, SYT11, NLGN1, LIN7C, SYT6, RPH3A, SSPN, GRM8, CHRNB3, SV2B, SV2A, DOC2B, APBA1
GO:0045596 ~ negative regulation of cell differentiation	19	0.01342	TWSG1, SIX2, SOCS5, JAG1, LIG4, GLI2, SOX9, ADIPOQ, SHH, HES1, NOTCH3, INHBA, RNF6, HOXA2, RHOA, TGFBR3, TOB2, BMPR1A, TOB1
IPR001565:Synaptotagmin	5	0.005111	SYT4, SYT11, SYT6, RPH3A, DOC2B
GO:0005544 ~ calcium-dependent phospholipid binding	4	0.115606	SYT11, ANXA1, DOC2B, RBM12
GO:0045664 ~ regulation of neuron differentiation	12	0.048765	NOTCH3, AMIGO1, HES1, HOXA2, CDK5R1, RNF6, NLGN1, RHOA, SEMA4D, GLI2, SHH, KALRN

**Table 2:** The significantly enriched GO terms associated with pain in sheep

Go term	Count	P value	Gene
Up-regulated			
GO:0015276 ~ ligand-gated ion channel activity	14	2.48E−05	GABRG3, SHROOM2, GRIK1, GABRB2, GLRA3, GABRB1, GRIN2A, GRIA4, CNGB3, KCNJ6, GRID2, CHRNA5, KCNH7, SCNN1B
GO:0006811 ~ ion transport	34	2.10E−03	SLC5A5, GRIK1, GABRB2, ATOX1, GABRB1, GLRA3, SLC39A12, ATP5G1, CNGB3, CHRNA5, GRID2, SLC30A3, SCNN1B, ATP5I, OCA2, SLC30A7, TRPM3, GABRG3, KCND2, KCNB2, SLC12A3, GRIN2A, GRIA4, CACNG2, TCN1, ATP13A4, SLCO1B3, KCNJ6, CATSPER3, KCNH7, HEPH, KCTD16, SLC13A4, CLCN7
GO:0022836 ~ gated channel activity	20	4.61E−04	GABRG3, SHROOM2, KCND2, GRIK1, GABRB2, KCNB2, GLRA3, GABRB1, GRIN2A, CACNG2, GRIA4, CNGB3, KCNJ6, CATSPER3, GRID2, CHRNA5, KCNH7, KCTD16, SCNN1B, CLCN7
GO:0005216 ~ ion channel activity	21	0.002572	TRPM3, GABRG3, SHROOM2, KCND2, GRIK1, GABRB2, KCNB2, GLRA3, GABRB1, GRIN2A, CACNG2, GRIA4, CNGB3, KCNJ6, CATSPER3, GRID2, CHRNA5, KCNH7, KCTD16, SCNN1B, CLCN7
GO:0060284 ~ regulation of cell development	4	0.895878	MUSK, MAP1B, NLGN1, SOX5
GO:0007268 ~ synaptic transmission	15	0.020093	GABRG3, KCND2, MYO6, GRIK1, GABRB2, GLRA3, PCDHB4, NLGN1, GRIN2A, GRIA4, MUSK, GRID2, CHRNA5, GAD1, NPFF
GO:0019226 ~ transmission of nerve impulse	16	0.033585	GABRG3, KCND2, MYO6, GRIK1, GABRB2, GLRA3, PCDHB4, NLGN1, GRIN2A, CACNG2, GRIA4, MUSK, GRID2, CHRNA5, GAD1, NPFF
GO:0006812 ~ cation transport	22	0.042508	TRPM3, SLC5A5, KCND2, KCNB2, SLC12A3, ATOX1, SLC39A12, GRIN2A, CACNG2, ATP5G1, TCN1, ATP13A4, KCNJ6, CATSPER3, KCNH7, HEPH, KCTD16, SLC30A3, SLC13A4, SCNN1B, ATP5I, SLC30A7
IPR001508:NMDA receptor	4	0.009544	GRIK1, GRID2, GRIN2A, GRIA4
GO:0005254 ~ chloride channel activity	5	0.116044	GABRG3, GABRB2, GABRB1, GLRA3, CLCN7
GO:0005244 ~ voltage-gated ion channel activity	9	0.134055	KCND2, KCNJ6, CATSPER3, KCNB2, GRIN2A, KCNH7, CACNG2, KCTD16, CLCN7
GO:0031404 ~ chloride ion binding	5	0.138619	GABRG3, GABRB2, GABRB1, GLRA3, CLCN7
Down-regulated			
GO:0005248 ~ voltage-gated sodium channel activity	9	2.43E−06	SCN2B, SCN3B, PKD2, SCN9A, SCN4B, SCN11A, SCN8A, SCN7A, SCN10A
GO:0001518 ~ voltage-gated sodium channel complex	7	6.00E−05	SCN2B, SCN9A, SCN4B, SCN11A, SCN8A, SCN7A, SCN10A
GO:0031402 ~ sodium ion binding	21	1.81E−04	SLC8A3, SLC13A5, PDXK, SCN2B, SLC12A2, SCN3B, ATP1B2, SLC9A3, KIAA1919, SLC34A2, SLC17A6, SLC6A8, SCN9A, SLC4A8, SCN4B, SLC13A3, SCN11A, SCN7A, SCN8A, HCN4, SCN10A
GO:0022843 ~ voltage-gated cation channel activity	19	0.015225	KCNJ16, KCNAB2, SCN2B, SCN3B, KCNA2, KCTD20, KCNJ5, KCTD9, KCNK6, SCN9A, PKD2, SCN4B, SCN11A, SCN7A, SCN8A, KCNQ2, HCN4, KCTD12, SCN10A
hsa04080:neuroactive ligand-receptor interaction	10	0.978027	GABRG2, GABRE, HTR1B, PTGER3, TRPV1, P2RY1, LTB4R2, AVPR1A, GABBR2, PTAFR
GO:0005083 ~ small GTPase regulator activity	36	4.43E−04	ALS2, TBC1D9, CYTH1, PREX1, MYO9B, CYTH3, ARFGEF2, RIMS1, PLEKHG3, PLEKHG2, DMXL2, PLEKHG7, SOS1, GOPC, ARHGAP1, TBC1D13, PLEKHG5, RAPGEF2, FGD5, ERRFI1, IQSEC1, TBC1D2B, ABR, TRIO, RPH3A, VAV2, ARHGEF10, MAP4K4, TBC1D24, CDC42BPG, ADAP2, SGSM1, SGSM2, CIT, CDC42BPB, RANBP10
GO:0008076 ~ voltage-gated potassium channel complex	7	0.526697	KCNJ5, KCTD9, KCNK6, KCNA2, KCTD20, KCNQ2, KCTD12
GO:0006952 ~ defense response	33	0.971848	ALS2, YWHAZ, C3, TRPV1, TLR2, AFAP1L2, CX3CL1, ITGB1, TLR7, IL31RA, FCN3, SCN9A, THBS1, MX2, FN1, SPP1, PTGER3, LIPA, IL1RL1, SAAL1, SOCS6, SMAD1, CD1D, DDX58, SIGLEC1, ABCC9, SARM1, CCR5, TFRC, CCR2, ICOSLG, CD14, PTAFR

We found that the DEGs only identified in goat, such as immunoglobulin superfamily member 6 (IGSF6), C-X-C motif chemokine ligand 11 (CXCL11), pentraxin 3 (PTX3), C-C motif chemokine ligand 17 (CCL17), and chemokine (C-C motif) receptor-like 1 (CCRL1) were significantly enriched in chemokine signaling pathway, chemotaxis, inflammatory response, cytokine–cytokine receptor interaction, and immune response (Additional file 1: Table S2), while the DEGs only identified in sheep, including colony-stimulating factor 2 (CSF2), CCAAT/enhancer binding protein gamma (CEBPG), RELB proto-oncogene, nuclear factor kappa B (NF-KB) subunit (RELB), B cell CLL/lymphoma 3 (BCL3), and integrin subunit beta 1 (ITGB1), were markedly enriched in intermediate filament, leukocyte differentiation, B cell activation, cellular response to stress, cytokine–cytokine receptor interaction, and cell–cell signaling (Additional file 1: Table S2).

We also performed GO analysis of co-DEGs between goat and sheep. The results showed that the upregulated co-DEGs were significantly enriched in embryonic organ development, transmembrane protein, cytoskeleton, neuroactive ligand-receptor interaction, and synapse, while the downregulated co-DEGs were enriched in cell adhesion, extracellular matrix, extracellular matrix-receptor interaction, cell motion, and signal peptide (Additional file 1: Table S3).

## Discussion

In the present study, we investigated the mechanism underlying the response to pain in goat and sheep through transcriptome sequencing using DRG tissues of CFA-induced sheep and goats and subsequent bioinformatics analysis. The results showed that the DEGs identified in CFA-treated goats, such as CCL27, GRIA2, and SCN3A, were mainly enriched in GO functions associated with NMDA receptor, inflammatory response, and immune response and may play key roles in the process of response to pain in goats. The DEGs identified in CFA-treated sheep, such as GABA-related DEGs (GABRG3, GABRB2, and GABRB1), SCN9A, and TRPV1, were mainly enriched in GO functions related to neuroactive ligand-receptor interaction, NMDA receptor, and defense response and may be involved in the process of response to pain in sheep. These data elucidated the possible mechanism involved in the process of response to pain in sheep and goat and may suggest potential drug targets for the treatment of chronic pain.

In a previous study, NMDA receptor was shown to play a key role in the neurophysiology of pain transmission and modulation [29]. In the insular cortex, the increase in synaptic NMDA receptors contributes to the development of neuropathic pain [30]. Wang et al. demonstrated that Ht31 peptide repressed inflammatory pain in mice by blocking the NMDA receptor-mediated nociceptive transmission [31]. Moreover, inflammatory response is also considered as the key mechanism involved in the regulation of neuropathic pain, and modulation of this process may serve as a treatment strategy for neuropathic pain [32]. The key DEGs associated with pain in goat were identified in the present study. CCL27 is a cytokine involved in chronic pain [33]. In a CFA-induced chronic inflammatory pain model, mice with null mutation of GRIA2 have increased thermal and mechanical hyperalgesia [34]. SCN3A is a voltage-gated sodium channel gene that regulates the important role of miR-30b in spinal nerve ligation-induced neuropathic pain in rats [35]. Given the key role of these GO functions and their enriched genes, we speculate that NMDA receptor and inflammatory response as well as the above mentioned enriched DEGs may be the key mechanisms that mediate the response to pain in goat.

We found that the NMDA receptor is a key GO function enriched by DEGs identified in CFA-treated sheep, indicative of its involvement in the process of response to pain in sheep. In addition, DEGs identified in CFA-treated sheep were mainly enriched in GO functions related to neuroactive ligand-receptor interaction. Sprangers et al. confirmed the association of neuroactive ligand-receptor interaction with disease-related pain and pain severity, duration, and relief [35], suggestive of the key role of this pathway in the process of response to pain. Moreover, GABA-related DEGs such as GABRG3, GABRB2, and GABRB1 were markedly upregulated. The inhibitory transmitter GABA has been shown to play a crucial role in the modulation of pain transmission [36, 37]. Gwak and Hulsebosch revealed that the spinal cord injury-induced hypofunction of GABAergic tone was involved in enhanced synaptic transmission, which results in neuronal hyperexcitability in dorsal horn neurons and subsequent chronic neuropathic pain [38]. These findings imply that the inhibitory transmitter GABA may serve as a key mediator involved in the response to pain in sheep. We also observed downregulation in SCN9A and TRPV1 expressions. Previous studies have confirmed that the mutations in SCN9A (NaV1.7) may cause pain disorders and pain sensitivity [39, 40]. In addition, TRPV1 is a molecular sensor of heat and capsaicin that was shown to regulate chronic pain by modulating central terminal sensitization [41], and targeting TRPV1 is considered as a promising strategy for the treatment of chronic pain [42]. Given the key role of SCN9A and TRPV1 in chronic pain modulation, we speculate that the downregulation of SCN9A and TRPV1 may contribute to the process of response to pain in sheep.

Some limitations of the present study include the small sample size owing to the limitation of experimental cost that may influence the stability of statistical power. In addition, the identified DEGs and pathways were not verified by functional experiments. Moreover, CFA is used as a co-adjuvant in antibody production. Whether CFA acts as an immunopotentiator and participates in the immune response to pain is unclear. Therefore, further investigations with additional experiments and high throughput data are warranted to confirm the findings of the present study.

## Conclusion

In conclusion, our data indicate that NMDA receptor, inflammatory response, and immune response as well as DEGs such as CCL27, GRIA2, and SCN3A may be the key factors that mediate the process of response to chronic pain in goats. Neuroactive ligand-receptor interaction and NMDA receptor as well as GABA-related DEGs (GABRG3, GABRB2, and GABRB1), SCN9A, and TRPV1 may be involved in the process of response to pain in sheep. Our findings may provide some drug targets to prevent chronic pain.

## Additional file

**Additional file 1: Table S1.** GO term analysis of differentially expressed genes between strains. **Table S2.** GO term analysis of differentially expressed genes only in sheep or goat. **Table S3.** GO term analysis of co-differentially expressed genes in sheep and goat.

## Abbreviations

CFA: complete Freund’s adjuvant
DRG: dorsal root ganglion
DEG: differentially expressed gene
GO: gene ontology
