# Supplementary material for: Identification of key genes and pathways involved in response to pain in goat and sheep by transcriptome sequencing
Source: Biol Res. 2018 Aug 17;51:25. doi: 10.1186/s40659-018-0174-7 (PMC6098572; doi:10.1186/s40659-018-0174-7)
Supplement: Supplementary file 1 — Additional file 1: Table S1. GO term analysis of differentially expressed genes between strains. Table S2. GO term analysis of differentially expressed genes only in sheep or goat. Table S3. GO term analysis of co-differentially expressed genes in sheep and goat. [file 40659_2018_174_MOESM1_ESM.docx]

**Additional file 1: Table S1** GO term analysis of differentially expressed genes between strains

| **Clusters** | **Treatment vs. control (goat)** | **Treatment vs. control (sheep)** |
| --- | --- | --- |
| Up-regulated | | |
| Cluster 1 | Low density lipoprotein-receptor, class A, cysteine-rich (Enrichment Score: 3.17) | Ligand-gated ion channel activity; ligand-gated channel activity; ion transport; cell junction; gated channel activity; transport; extracellular ligand-gated ion channel activity; ionic channel; Neuroactive ligand-receptor interaction; postsynaptic cell membrane; substrate specific channel activity; synapse; neurotransmitter receptor; synaptic transmission; Neurotransmitter-gated ion-channel; cell-cell signaling; transmission of nerve impulse; GABA-A receptor activity; neurotransmitter receptor activity; chloride channel activity; chloride channel; chloride transport; anion binding; neurological system process (Enrichment Score: 1.93) |
| Cluster 2 | Glycoprotein; signal; signal peptide; disulfide bond; extracellular region; secreted (Enrichment Score: 2.66) | Extracellular ligand-gated ion channel activity; postsynaptic cell membrane; Glutamate receptor, L-glutamate/glycine-binding; NMDA receptor; Glutamate receptor-related; Ionotropic glutamate receptor; ionotropic glutamate receptor activity; extracellular-glutamate-gated ion channel activity; glutamate signaling pathway; Extracellular ligand-binding receptor; regulation of excitatory postsynaptic membrane potential; regulation of postsynaptic membrane potential; regulation of membrane potential (Enrichment Score: 1.69) |
| Cluster 3 | Glycoprotein; transmembrane;  transmembrane region; integral to membrane; intrinsic to membrane; membrane (Enrichment Score: 2.23) | Multicellular organism reproduction; reproductive process in a multicellular organism; gamete generation; sexual reproduction; spermatogenesis; male gamete generation; spermatogenesis (Enrichment Score: 1.51) |
| Cluster 4 | Ig-like 1; Ig-like 2; Ig-like 3 (Enrichment Score: 2.11) | Secondary metabolic process; melanin biosynthetic process; melanin metabolic process; Albinism; pigmentation; pigment biosynthetic process; pigment metabolic process; pigmentation during development (Enrichment Score: 1.43) |
| Cluster 5 | Plasma membrane part; integral to plasma membrane; intrinsic to plasma membrane; plasma membrane; cell membrane (Enrichment Score: 1.95) | Mitochondrial electron transport, NADH to ubiquinone; respiratory chain; oxidative phosphorylation; ATP synthesis coupled electron transport; mitochondrial ATP synthesis coupled electron transport; mitochondrion inner membrane; respiratory electron transport chain; Huntington's disease; ubiquinone; Parkinson's disease; energy derivation by oxidation of organic compounds; electron transport; cellular respiration; electron transport chain; generation of precursor metabolites and energy; mitochondrion; oxidation reduction (Enrichment Score: 1.42) |
| Cluster 6 | TSP type-1; Thrombospondin, type 1 repeat; Peptidase M12B, ADAM-TS (Enrichment Score: 1.88) | Membrane; transmembrane; topological domain:Cytoplasmic; glycoprotein; integral to membrane; intrinsic to membrane (Enrichment Score: 1.40) |
| Cluster 7 | Nitrogen metabolism; Carbonic anhydrase, alpha-class, conserved site; carbonate dehydratase activity; carbonate dehydratase; lyase; hydro-lyase activity; one-carbon metabolic process (Enrichment Score: 1.87) | Flagellum part; microtubule-based flagellum part; microtubule-based flagellum; flagellum (Enrichment Score: 1.37) |
| Cluster 8 | Ion transport; substrate specific channel activity; gated channel activity; ligand-gated channel activity; ligand-gated ion channel activity; channel activity; passive transmembrane transporter activity; ion channel activity; transport; transmembrane transport; cation channel activity; metal ion transport; metal ion transmembrane transporter activity; cation transport; voltage-gated channel; alkali metal ion binding; potassium channel; potassium transport; potassium (Enrichment Score: 1.73) | BRIGHT; AT-rich interaction region (Enrichment Score: 1.29) |
| Cluster 9 | Anion transmembrane transporter activity; anion transport; organic anion transport (Enrichment Score: 1.68) | Embryonic organ morphogenesis; ear development; embryonic organ development; ear morphogenesis; sensory organ development; inner ear development; inner ear morphogenesis; embryonic morphogenesis; embryonic development ending in birth or egg hatching; chordate embryonic development (Enrichment Score: 1.29) |
| Cluster 10 | Vision; sensory perception of light stimulus; visual perception; retinitis pigmentosa; neurological system process; sensory perception; cognition; sensory transduction (Enrichment Score: 1.63) | Developmental maturation; cell maturation; oocyte maturation; germ cell development; oocyte development; oocyte differentiation; reproductive cellular process; oogenesis (Enrichment Score: 1.29) |
| Down-regulated | | |
| Cluster 1 | Signal; glycoprotein; signal peptide; extracellular region part; secreted; extracellular region; disulfide bond; extracellular space (Enrichment Score: 8.24) | Cell adhesion; biological adhesion; Protocadherin gamma; Cadherin, N-terminal; homophilic cell adhesion; Cadherin (Enrichment Score: 7.60) |
| Cluster 2 | Extracellular matrix; proteinaceous extracellular matrix; extracellular region part; extracellular matrix part  (Enrichment Score: 8.20) | Metal ion binding; ion binding; cation binding; zinc-finger; zinc ion binding; transition metal ion binding (Enrichment Score: 6.83) |
| Cluster 3 | Glycosaminoglycan binding; polysaccharide binding; pattern binding; heparin binding; carbohydrate binding (Enrichment Score: 6.64) | Pleckstrin homology; Pleckstrin homology-type (Enrichment Score: 4.25) |
| Cluster 4 | Cell adhesion; biological adhesion (Enrichment Score: 5.33) | Cell junction; synapse; synapse part (Enrichment Score: 4.12) |
| Cluster 5 | Skeletal system development; bone development; ossification; osteoblast differentiation (Enrichment Score: 5.29) | BTB; Kelch related (Enrichment Score: 3.62) |
| Cluster 6 | Plasma membrane part; integral to plasma membrane; intrinsic to plasma membrane (Enrichment Score: 4.17) | Membrane; glycoprotein; transmembrane region; transmembrane; integral to membrane; intrinsic to membrane (Enrichment Score: 3.60) |
| Cluster 7 | Metal ion-binding site: Calcium (Enrichment Score: 4.11) | Fibronectin type-III (Enrichment Score: 3.43) |
| Cluster 8 | Respiratory system development; lung development; respiratory tube development; tube development (Enrichment Score: 3.97) | Cellular component morphogenesis; cell morphogenesis; cell projection morphogenesis; cell part morphogenesis; neuron projection morphogenesis; cell morphogenesis involved in neuron differentiation; neuron projection development; cell projection organization; axonogenesis; neuron development; neuron differentiation (Enrichment Score: 3.93) |
| Cluster 9 | Collagen; collagen triple helix repeat; hydroxylation (Enrichment Score: 3.49) | Cytoskeletal protein binding; actin cytoskeleton; actin-binding (Enrichment Score: 2.88) |
| Cluster 10 | Membrane; transmembrane; transmembrane region; intrinsic to membrane; integral to membrane (Enrichment Score: 3.25) | C2 calcium-dependent membrane targeting; C2 membrane targeting protein; Synaptotagmin (Enrichment Score: 2.80) |

**Additional file 1: Table S2** GO term analysis of differentially expressed genes only in sheep or goat

| **Clusters** | **Goat** | **Sheep** |
| --- | --- | --- |
| Cluster 1 | Leucine-rich repeat (Enrichment Score: 3.77) | Intermediate filament; keratin epidermis development; ectoderm development; compositionally biased region:Gly-rich; epithelium development; epithelial cell differentiation (Enrichment Score: 2.78) |
| Cluster 2 | Signal; signal peptide; secreted; extracellular region; disulfide bond (Enrichment Score: 2.84) | Myeloid cell differentiation; hemopoietic or lymphoid organ development; immune system development; hemopoiesis; erythrocyte differentiation; erythrocyte homeostasis; homeostasis of number of cells; leukocyte differentiation; myeloid leukocyte differentiation; B cell differentiation; lymphocyte differentiation; cell activation; B cell activation; leukocyte activation; lymphocyte activation (Enrichment Score: 2.21) |
| Cluster 3 | Fibrinogen, alpha/beta/gamma chain, C-terminal globular, subdomain 1 (Enrichment Score: 1.71) | Metal ion-binding site:Calcium; via 3-oxoalanine; sulfuric ester hydrolase activity; sulfuric ester hydrolase (Enrichment Score: 2.17) |
| Cluster 4 | Extracellular region part; proteinaceous extracellular matrix extracellular matrix (Enrichment Score: 1.71) | Myeloid cell differentiation; cell maturation; developmental maturation (Enrichment Score: 1.91) |
| Cluster 5 | Phosphoric diester hydrolase activity; phospholipase activity; lipase activity (Enrichment Score: 1.14) | Protein dimerization activity; protein homodimerization activity; identical protein binding (Enrichment Score: 1.20) |
| Cluster 6 | Response to wounding; cytokine; chemokine signaling pathway; chemotaxis; inflammatory response; taxis; cytokine-cytokine receptor interaction; immune response; locomotory behavior; inflammatory response; defense response; behavior; regulation of cell proliferation; cell-cell signaling (Enrichment Score: 0.96) | Stress-activated protein kinase signaling pathway; protein kinase cascade; MAPKKK cascade; JNK cascade; cellular response to stress; intracellular signaling cascade (Enrichment Score: 0.98) |
| Cluster 7 | Transcriptional regulator SCAN (Enrichment Score: 0.93) | Iron; iron-sulfur; oxidation reduction; oxidoreductase (Enrichment Score: 0.92) |
| Cluster 8 | Fibronectin, type III (Enrichment Score: 0.91) | Protein import; cellular protein localization; cellular macromolecule localization; protein localization in organelle; protein targeting; intracellular protein transport; intracellular transport; protein localization; nucleocytoplasmic transport; nuclear transport; protein import into nucleus; nuclear import; establishment of protein localization; protein localization in nucleus; protein transport  (Enrichment Score: 0.92) |
| Cluster 9 | Regulation of phospholipase activity; regulation of lipase activity; regulation of hydrolase activity (Enrichment Score: 0.79) | Chemokine signaling pathway; locomotory behavior; Cytokine-cytokine receptor interaction; immune response; chemotaxis; taxis; behavior; cell-cell signaling (Enrichment Score: 0.86) |
| Cluster 10 | Oxidation reduction (Enrichment Score: 0.76) | Alcohol catabolic process; cellular carbohydrate catabolic process; carbohydrate catabolic process; glucose catabolic process; cellular carbohydrate biosynthetic process; hexose catabolic process; monosaccharide catabolic process; glucose metabolic process; carbohydrate biosynthetic process; hexose metabolic process; monosaccharide metabolic process (Enrichment Score: 0.84) |

**Additional file 1: Table S3** GO term analysis of co-differentially expressed genes in sheep and goat

| **Clusters** | **Up-regulated** | **Down-regulated** |
| --- | --- | --- |
| Cluster 1 | Cell projection; flagellum (Enrichment Score: 1.84) | Cell adhesion; biological adhesion; Cadherin; homophilic cell adhesion (Enrichment Score: 3.36) |
| Cluster 2 | mRNA metabolic process; RNA splicing; RNA processing; mRNA processing (Enrichment Score: 1.27) | BTB; Kelch related (Enrichment Score: 3.28) |
| Cluster 3 | mRNA metabolic process; mRNA transport; nucleic acid transport; RNA transport; establishment of RNA localization; RNA localization; nucleobase, nucleoside, nucleotide and nucleic acid transport (Enrichment Score: 1.18) | Extracellular matrix; extracellular region; extracellular space (Enrichment Score: 2.70) |
| Cluster 4 | mRNA metabolic process; mRNA catabolic process; RNA catabolic process; cellular macromolecule catabolic process; macromolecule catabolic process (Enrichment Score: 1.18) | ECM-receptor interaction; heparin-binding; Laminin G, thrombospondin-type, N-terminal; Focal adhesion (Enrichment Score: 2.44) |
| Cluster 5 | Embryonic organ morphogenesis; ear morphogenesis; embryonic organ development; inner ear development; sensory organ development; chordate embryonic development; embryonic development ending in birth or egg hatching (Enrichment Score: 1.07) | Heparin-binding; EGF; cell migration; localization of cell; cell motility; cell motion (Enrichment Score: 2.20) |
| Cluster 6 | Transmembrane protein; hemopoiesis; hemopoietic or lymphoid organ development; hemopoietic or lymphoid organ development; immune system development (Enrichment Score: 0.99) | Signal; signal peptide; glycoprotein; disulfide bond (Enrichment Score: 2.15) |
| Cluster 7 | Cytoskeleton; microtubule; cytoskeletal part (Enrichment Score: 0.99) | von Willebrand factor, type C; Thrombospondin, type 1 repeat (Enrichment Score: 1.80) |
| Cluster 8 | Immunoglobulin-like (Enrichment Score: 0.87) | Short sequence motif:Cell attachment site; platelet alpha granule lumen; cytoplasmic membrane-bounded vesicle lumen; vesicle lumen; ~cytoplasmic vesicle;collagen binding; cytoplasmic vesicle part (Enrichment Score: 1.58) |
| Cluster 9 | Neuroactive ligand-receptor interaction; synapse; postsynaptic cell membrane; cell junction; anion transport; transport; ion transport; ionic channel (Enrichment Score: 0.87) | Extracellular matrix part; collagen; basement membrane; Collagen triple helix repeat; extracellular matrix organization; extracellular structure organization (Enrichment Score: 1.43) |
| Cluster 10 | Gamete generation; sexual reproduction; spermatogenesis; male gamete generation; multicellular organism reproduction; reproductive process in a multicellular organism (Enrichment Score: 0.83) | Growth factor binding; transforming growth factor beta binding; cytokine binding (Enrichment Score: 1.42) |
